# Exploratory drive, fear, and anxiety are dissociable and independent components in foraging mice

**DOI:** 10.1038/s41398-021-01458-9

**Published:** 2021-05-26

**Authors:** Daniel E. Heinz, Vivian A. Schöttle, Paulina Nemcova, Florian P. Binder, Tim Ebert, Katharina Domschke, Carsten T. Wotjak

**Affiliations:** 1 Max Planck Institute of Psychiatry, Research Group Neuronal Plasticity, Kraepelinstr. 2-10, 80804 Munich, Germany; 2 Max Planck School of Cognition, Stephanstr. 1A, 04103 Leipzig, Germany; 3 Department of Psychiatry and Psychotherapy, Medical Center - University of Freiburg, Faculty of Medicine, University of Freiburg, Hauptstr. 5, 79104 Freiburg, Germany; 4 Max Planck Institute of Psychiatry, Department of Translational Psychiatry, Kraepelinstr. 2-10, 80804 Munich, Germany; 5 Boehringer Ingelheim Pharma & Co KG, Central Nervous System Diseases Research (CNSDR), Birkendorfer Straße 65, 88397 Biberach an der Riss, Germany

**Keywords:** Neuroscience, Physiology, Psychology

## Abstract

Anxiety-like behavior of rodents is frequently accompanied by reduced exploration. Here, we identify dissociable components of anxiety, fear, and exploratory drive of sated and foraging mice. With the help of behavioral assays, including the open field task, elevated plus maze, dark–light transition task, and beetle mania task, we demonstrate a general increase in exploration by food restriction. Food-restricted mice bred for high anxiety behavior (HAB) showed ameliorated anxiety- but not fear-related behavior. By means of principal component analysis, we identified three independent components, which resemble the behavioral dimensions proposed by Gray’s Reinforcement Sensitivity Theory (approach behavior, avoidance behavior, and decision making). Taken together, we demonstrate anxiolytic consequences of food restriction in a mouse model of anxiety disorders that can be dissociated from a general increase in foraging behavior.

## Introduction

Animals constantly take decisions, whenever they interact with their environment^1,2^. Such decisions depend on motivational states. In the absence of motivational conflicts, animals approach pleasant but avoid unpleasant situations. Quite often, however, they have to resolve conflicts between opposing motivations, such as foraging for food, shelter, or mating partners in potentially unsecure environments on the one, and staying in their comfort zones on the other hand side. Resolution of such conflicts seems to be key to anxiety^1,3^. Accordingly, several experimental paradigms provide the animals with the choice between unprotected vs. protected areas (e.g., center vs. border of an open field, open vs. closed arms of an elevated plus maze (EPM), and light vs. dark compartment in the light–dark box^4,5^), in order to measure anxiety-related behavior. Most studies carefully contrast between unpleasant/dangerous vs. pleasant/safe conditions (i.e., the avoidance component), without paying similar attention to interindividual differences in exploratory drive (i.e., the approach component). In consequence, high levels of avoidance might be mistaken for high levels of anxiety if animals lack motivation to explore unsecure places and, in consequence, an inner conflict between opposing motivations. For instance, rats and mice show less exploration of the open arms of an EPM upon repeated testing. This increase in avoidance behavior is insensitive to the treatment with anxiolytic compounds^6^, and, thus, does not reflect higher levels of anxiety, but the lack of motivation for open arm exploration once the animals have learned about the nonavailability of food, shelter, or mating partners. Therefore, it is essential to dissect exploratory behavior into its different motivational components and conflict-solving measures.

The Reinforcement Sensitivity Theory proposed by Gray^7,8^ and extended by others^9–12^ provides a theoretical framework for the description of interindividual differences in approach and avoidance behavior. It suggests the existence of three neuronal networks (dimensions) accounting for exploratory drive/approach behavior (Behavioral Activation System, BAS), avoidance behavior (Fight Flight Freeze System, FFFS), and conflict solving (Behavioral Inhibition System, BIS)^13^. Together, this provides a sophisticated model of emotion, motivation, personality, and psychopathology^11^.

So far, however, this theory has received little attention in animal studies on anxiety-related behavior. Therefore, the current study set out to validate this theoretical framework exemplarily for mice, which were selectively bred for high levels of open arm avoidance on the EPM (high anxiety behavior mice, HAB^14^) in comparison to less avoidant controls (normal anxiety behavior mice, NAB). We systematically changed the motivational state of the animals^15^ by comparing food restricted with ad libitum-fed mice and studied consequences on foraging behavior in a battery of conflict (open field, EPM, and light–dark avoidance) and non-conflict tasks (i.e., in response to an erratically moving robo-beetle as a potentially threatening stimulus; beetle mania task (BMT)^16^). With the help of exploratory statistics, we were able to reduce the dimensions of the manifold behavioral measures to three main components, which seem to be controlled by the BAS, FFFS, and BIS. This way we could dissociate motivational consequences of food restriction on foraging behavior from its anxiolytic impact. To exclude that differences in the behavioral consequences of food restriction between NAB and HAB mice simply resulted from differences in motivational changes, we additionally tested the two lines in an effort-related operant conditioning task, in which the animals had to work for food.

## Materials and methods

### Animals

Male HAB (*N* = 36) and NAB (*N* = 33) mice were bred at the Max Planck Institute of Biochemistry, Martinsried, Germany. The two lines originated from selective breeding of outbred CD1 mice based on their behavioral performance on the EPM^14^. Experiments were conducted at an age between 3 and 5 months at the Max Planck Institute of Psychiatry, Munich, Germany. Starting at least 10 days before the experiment, mice were kept singly in order to control individual food intake. They were housed in IVC cages (individually ventilated cages type 2, green line; Tecniplast, Hohenpeißenberg, Germany) equipped with bedding and rodent tunnel (4.5 × 4 cm, diameter: 30 mm; ABEDD, Vienna, Austria) under a 12 h light–dark cycle (lights off: 7 p.m.) with ad libitum access to water and food (in case of nonfood restricted controls) or restricted food supply. All animal studies were conducted in accordance with the recommendations of the Federation for Laboratory Animal Science Associations and were approved by the Government of Upper Bavaria (AZ: 55.2-2532.Vet_02-17-171).

### Food restriction (FR)

Body weight (BW) was measured on three consecutive days. Based on their individual baseline BW, we supplied the mice with limited amounts of food (Altromin Haltungsdiät 1328; Altromin Spezialfutter, Lage, Germany) to maintain them at ~85% of the baseline over the course of experiments. On experimental days, we weighed the animals before and supplied them with food after the experiments, at the end of the light/beginning of dark phase.

### Behavioral tests

Except for the operant conditioning experiments (“Operant conditioning” section), experiments were performed during the inactive phase of the circadian rhythm. The behavioral setups were placed in observational areas in an experimental room, which was connected to the holding room by a door. The observational areas were separated from the rest of the experimental room by black walls and curtains. For each trial, mice were transported from the holding to the dimly lit experimental room and individually placed into the setups. The setups were cleaned with water containing detergent between the trials and carefully dried. Experiments were performed and the behavior was scored by experimenters unaware of the experimental conditions.

#### Open field test (OFT)

Mice were placed into a white PVC box (L40 × W40 × H40 cm; illumination: <25 Lux) facing the wall. They were allowed to explore the arena for 15 min. The arena was split into two virtual zones (outer zone: 10 cm away from the walls, inner zone: remaining part of 20 × 20 cm), and we used ANY-maze (4.99, Stoelting CO., USA) to automatically assess the following parameters: time in zones, distance in zones, and latency until the first entry into the center zone. Total distance moved was analyzed in 5-min bins. We recorded the videos for subsequent analysis of risk assessment (stretch-attend posture, SAPs) and rearing by an experimenter blind to the experimental conditions.

#### Elevated plus maze

The EPM consisted of four arms (L27.5 × W6 cm) that were arranged as a plus and connected by a central area (L6 × W6 cm). Two opposing arms contained side and end walls (H14.5 cm), whereas the two other arms were only engulfed by a 0.5 cm high rim. The maze was elevated above the floor (30.5 cm) and illuminated with low light (<25 Lux). In the beginning of the experiment, mice were placed in the closed arms facing the end wall and allowed to explore the maze for 15 min. ANY-maze was used for automated tracking of time and distance, and recording for subsequent manual scoring of latency to first open arm entry, time of SAPs, rearing, and head dipping events.

#### Dark–light transition task (DaLi)

The DaLi (also known as the light–dark box) apparatus consisted of two compartments, which were made out of black (W21 × L16 × H25 cm; illumination: <25 Lux) or white PVC (W21 × L30 × H25 cm; illumination: 300 Lux) and connected by a small opening (W6.5 × H10). Mice were placed into the dark compartment facing the wall and allowed to explore the box for 10 min. Videos were recorded using ANY-maze, video analyses of time and full step-out latency to the lit compartment (with all four paws) was assessed by an experimenter blind to the experimental conditions.

#### Beetle mania task

We used the BMT to assess active fear responses^16^. In brief, mice were allowed to acclimatize to the new environment for 5 min, whereby they were started at one end of the empty arena (gray PVC, L100 × W15 × H37 cm, equally divided into four virtual segments; illuminated with <25 Lux). During this habituation period, an experienced observer who was unaware of the mouse line and feeding status scored vertical (number of rearings) and horizontal (latency until exploration of the other end of the arena) exploration. Thereafter, the mice remained in the arena, and we confronted them with an erratically moving robo-beetle (Hexbug Nano, Innovation First Labs Inc., Greenville, TX, USA; L4.5 × W1.5 × H1.8 cm, weight: 7.3 g, mean speed: 25 cm/s) for 5 min. The following behavioral parameters were scored online: total contacts (number of physical contacts between robo-beetle and mouse), tolerance upon contact (frequency of ignorance of the approaching robo-beetle, expressed as a percentage of total contacts), avoidance behavior upon contact (frequency of withdrawals from the beetle with accelerated speed, expressed as a percentage of total contacts), and the number of close following events (whereby the experimental subject was following the robo-beetle in close contact).

#### Operant conditioning

Food-restricted mice from a new batch of animals were trained using a Bussey-Saksida Rodent Operant Touchscreen Testing System (Campden Instruments Ltd., Loghborough, UK), operated by Whisker Server Version 4.6.2 (Cambridge University Technical Services Ltd., Cambridge, United Kingdom) and ABETII Touch Version 2.18 (Layafette Instrument Company, Lafayette, United States) essentially as described^17,18^. Task schedules, analysis scripts, and manuals were purchased (ABETII Touch Mouse Task for Progressive Ratio, Campden Instruments Ltd., Loughborough, United Kingdom). An initial habituation procedure, including reward (minimizing neophobia^17,19^) and chamber habituation, was followed by the initial touch training, whereby mice learned that a targeted nose poke to the presented stimulus results in reward delivery, indicated by an acoustic signal. After completing 30 trials within 60 min, mice were trained in fixed ratio (30 trials, 60 min; constant number of nose pokes required: 1, 3, or 5 times (for a minimum of 2 days until criteria were met), in order to obtain a single food reward. After successful completion of the fixed ratio 5 protocol at a ratio of 3:1 (correct:blank touches), we assessed the willingness to work for food by means of a progressive ratio protocol (PR). In this task, the animals could earn several rewards within a 1-h session, whereby they had to spend increasing effort in order to obtain food: the number of nose pokes to perform in order to obtain a single reward increased by four from trial to trials (PR4), thus resulting in a series of 1, 5, 9, 13... nose pokes. The trial in which the animals were not motivated to perform the required number of nose pokes increased by four anymore defined the breaking point.

Mice were excluded from the experiment if they remained within the same training stage for 25 days (four NAB and one HAB were excluded). PR4 took place on three consecutive days, followed by three days of fixed ratio 5 and three additional days of PR4. Trials to criterion (fixed ratio 1 until start PR), breaking point (last achieved stage of PR4), and target touches (total number of correct nose pokes until breaking point) were assessed and averaged over the six PR sessions.

### Experimental design

**Fig. 1:**
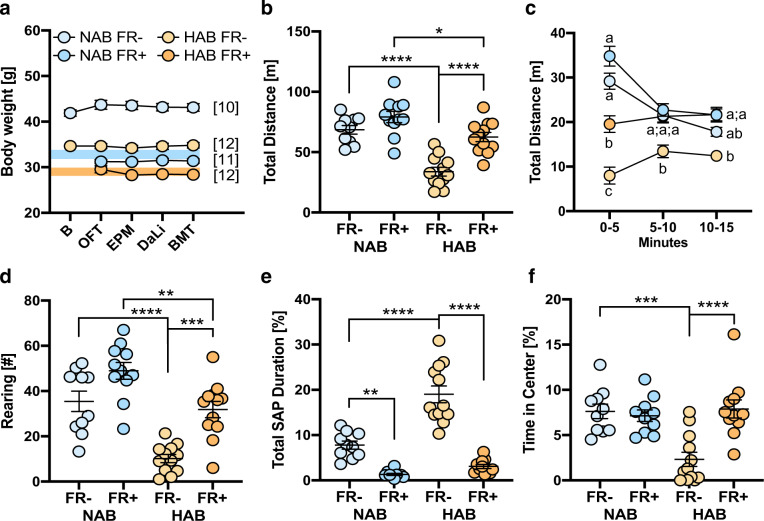
Food restriction procedure and open field test (OFT). **a** HAB and NAB mice were either food restricted (FR+) to reach 85% (shaded area) of their original body weight (baseline, B) or continued to be fed ad libitum (FR−). The body weight remained fairly stable throughout the entire test battery comprised by open field test (OFT), elevated plus maze test (EPM), dark–light transition task (DaLi), and beetle mania test (BMT), with at least 7 days of recovery between two subsequent tasks. Numbers in brackets: sample sizes. **b**–**f** Behavioral performance during a 15-min exposure to the OFT. Mean ± SEM with individual data. *p < 0.05, **p < 0.01, ***p < 0.001, ****p < 0.0001 (two-way ANOVA followed by Tukey’s post hoc test); in **c**, data points without shared letters are statistically significantly different (*p* < 0.05; three-way ANOVA for repeated measures, followed by Tukey’s post hoc test).

Experiments were performed with two independent groups of animals: HAB and NAB mice of the first batch were food restricted (FR+) or fed ad libitum (FR−), and subsequently tested in OFT, EPM, DaLi, and BMT (cf. Fig. 1a) with at least 7 days of recovery between two tests. The sample sizes were as follows: HAB FR+ = 12; HAB FR− = 12; NAB FR+ = 11; NAB FR− = 10. HAB and NAB mice of the second batch were all food restricted (FR+) and subjected to operant conditioning. The sample sizes were as follows: HAB FR+ = 12; NAB FR+ = 12.

### Statistical analysis

Data were processed using Microsoft Excel (v16.45) and analyzed and presented as individual data with means ± SEM (or median, if appropriate), using GraphPad Prism 8 (8.3.0). Unpaired *t* test, one-way analysis of variance (ANOVA), two-way ANOVA, or three-way ANOVA for repeated measures, followed by Tukey’s post hoc test (cf. figures). Differences were regarded as statistically significant if *p* < 0.05.

#### Principal component analysis (PCA)

We applied a theory- and data-driven approach to identify latent variables behind the readouts of all tasks. Following Gray’s Reinforcement Sensitivity Theory, we fixed the number of components of interest to three. We wanted to know from the data what these components look like. We performed a PCA in Matlab R2020a (MathWorks, Natick, MA, USA) on the *z*-scores of the 18 behavioral readouts and took the first three components. In order to improve interpretability, we applied a varimax rotation. Next, we calculated individual scores for each mouse and component by summing up the products between the readouts of the mouse and the loadings of the component.

## Results

### Food restriction (FR+)

Ad libitum-fed (FR−) HAB mice (mean: 34.6 ± 0.3 g) were significantly leaner than NAB mice (mean: 41.9 ± 0.7; *t*(43) = 9.95, *p* < 0.0001). Food restriction resulted in a reduction of the initial BW to ~80% in NAB and HAB, which remained stable over the course of the experiment (Fig. 1a).

### Open field test (OFT)

In terms of horizontal locomotor activity, two-way ANOVA (strain, FR) revealed significant main effects of *strain* (*F* (1, 41) = 42.32, *p* < 0.0001) and FR (*F* (1, 41) = 24.85, *p* < 0.0001), as well as a significant factorial interaction (*F* (1, 41) = 5.24, *p* = 0.0273). HAB FR− moved significantly less compared to NAB FR− mice, thus corroborating previous data^20^. Food restriction caused a strong increase in horizontal locomotion in HAB, but not NAB (Fig. 1b). These effects became evident from the beginning of the 15-min exposure (strain × FR × time interaction: *F* (6, 82) = 8.99, *p* < 0.0001) with HAB FR+, but not HAB FR−, reaching the level of performance of NAB FR− and NAB FR+ after 5 min (Fig. 1c).

Food restriction also caused a significant increase in vertical exploration (i.e., rearing; strain: *F* (1, 41) = 37.87, *p* < 0.0001; FR: *F* (1, 41) = 26.01, *p* < 0.0001), this time, however in both HAB and NAB mice (strain × FR: *F* (1, 41) = 1.41, *p* = 0.2413; Fig. 1d). Under basal conditions, HAB FR− mice were longer engaged in risk assessment (Fig. 1e) and spent less time in the center of the open field than NAB FR− mice (strain: *F* (1, 39) = 7.55, *p* = 0.0091; FR: *F* (1, 39) = 9.46, *p* = 0.0038; strain × FR: *F* (1, 39) = 13.31, *p* = 0.0008), indicative of their anxiety trait (Fig. 1f). Food restriction led to a decrease in risk assessment in both NAB and HAB (FR: *F* (1, 39) = 94.93, *p* < 0.0001; strain × FR: *F* (1, 39) = 16.94, *p* = 0.0002), and increased center time specifically in HAB FR+ (FR: *F* (1, 39) = 7.546, *p* = 0.0091; strain × FR: *F* (1, 39) = 13.31, *p* = 0.0009; Fig. 1f).

### Elevated plus maze

**Fig. 2:**
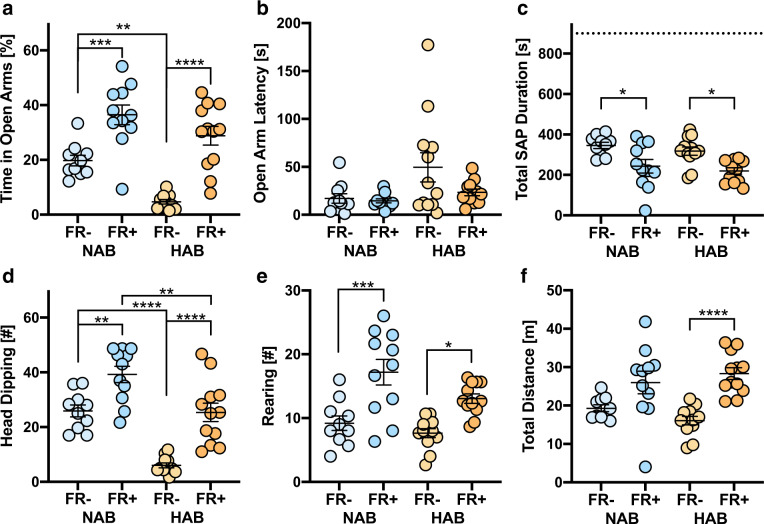
Elevated plus maze (EPM). **a**–**f** Behavioral performance of food-restricted (FR+) and ad libitum-fed (FR−) HAB and NAB mice during a 15-min exposure to the EPM. SAP stretch-attend postures. For further details, see Fig. 1.

In confirmation of the selective breeding strategy^14^, HAB FR− spent significantly less time on the open arms than NAB FR− (Fig. 2a), which was not reflected by significant differences in latency to the first open arm entry (Fig. 2b). Food restriction significantly increased the open arm time in both strains (FR: *F* (1, 40) = 54.77, *p* < 0.0001; strain × FR: *F* (1, 40) = 1.18, *p* = 0.1891; Fig. 2a), with HAB FR+ approaching the levels of performance of NAB FR+. Conversely, food restriction in general resulted in reduced risk assessment (strain: *F* (1, 41) = 1.29, *p* = 0.2621; FR: *F* (1, 41) = 19.72, *p* < 0.0001; strain × FR: *F* (1,41) = 0.01, *p* = 0.9178; Fig. 2c). At the same time, there was a general increase in exploratory behavior, which became evident from the increase in head dipping (strain: *F* (1, 40) = 41.87, *p* < 0.0001; FR: *F* (1, 40) = 39.24, *p* < 0.0001; strain × FR: *F* (1, 40) = 1.33, *p* = 0.2552; Fig. 2d), vertical (strain: *F* (1, 41) = 5.38, *p* = 0.0254; FR: *F* (1, 41) = 29.88, *p* < 0.0001; strain × FR: *F* (1, 41) = 1.10, *p* = 0.3013; Fig. 2e), and horizontal exploration (strain: *F* (1,41) = 0.06, *p* = 0.8027; FR: *F* (1, 41) = 27.42, *p* < 0.0001; strain × FR: *F* (1, 41) = 2.32, *p* = 0.1347; Fig. 2f).

### Dark–light transition task

**Fig. 3:**
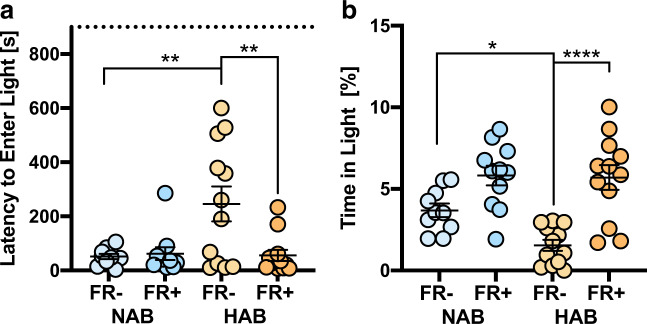
Dark–light transition task (DaLi). **a**, **b** Behavioral performance of food-restricted (FR+) and ad libitum-fed (FR−) HAB and NAB mice during a 15-min exposure to the DaLi setup. For further details see Fig. 1.

Also in the DaLi, food restriction reverted the anxiogenic phenotype of HAB FR− mice by reducing the latency to enter the light compartment (strain: *F* (1, 41) = 5.967, *p* = 0.0190; FR: *F* (1,41) = 5.48, *p* = 0.0242; strain × FR: *F* (1,41) = 6.82, *p* = 0.0125; Fig. 3a) and the time spent in the light compartment (strain: *F* (1,41) = 4.00, *p* = 0.0521; FR: *F* (1, 41) = 30.93, *p* < 0.0001; strain × FR: *F* (1, 41) = 3.18, *p* = 0.0822; Fig. 3b).

### Beetle mania task

**Fig. 4:**
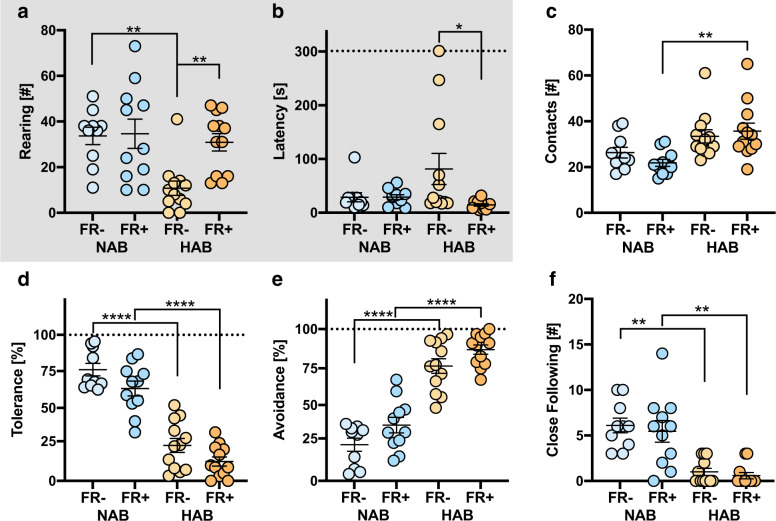
Beetle mania task (BMT). **a**, **b** Vertical (number of rearings) and horizontal exploration (latency until the animals have explored the other end of the 1 m arena) during the 5-min baseline exposure to the BMT setup without robo-beetle. **c**–**f** Behavioral responses to an erratically moving robe-beetle. Data shown in **d** and **e** were normalized to the number of contacts (**c**). For further details, see Fig. 1.

In agreement with the finding from the OFT, food restriction restored vertical (strain: *F* (1, 41) = 9.03, *p* = 0.0045; FR: *F* (1, 41) = 5.652, *p* = 0.0222; strain × FR: *F* (1, 41) = 4.69, *p* = 0.0361; Fig. 4a) and horizontal (strain: *F* (1, 41) = 1.41, *p* = 0.2425; FR: *F* (1, 41) = 4.26, *p* = 0.454; strain × FR: *F* (1. 41) = 4.36, *p* = 0.0431; Fig. 4b) exploration in HAB FR+, during the 5-min baseline before confrontation with the robo-beetle. During the subsequent exposure to the robo-beetle, HAB mice in general had significantly more contacts with the potentially threatening stimulus than NAB mice (strain: *F* (1, 41) = 14.35, *p* = 0.0005; FR: *F* (1, 41) = 0.16, *p* = 0.6888; strain × FR: *F* (1,41) = 1.48, *p* = 0.2308; Fig. 4c). Therefore, to facilitate between-line comparisons, we normalized tolerance and avoidance behavior (i.e., passive vs. active coping) to the number of confrontations. In agreement with previous observations^16^, HAB mice showed little tolerance (Fig. 4d) but exaggerated avoidance of the robo-beetle (Fig. 4e) compared to NAB mice (strain: *F* (1, 41) = 134.80, *p* < 0.0001). Importantly, food restriction caused a general decrease in tolerance (FR *F* (1, 41) = 7.44, *p* = 0.0094; strain × FR: *F* (1, 41) = 0.04, *p* = 0.8340; Fig. 4d) and an increase in avoidance (FR *F* (1, 41) = 7.35, *p* = 0.0098; strain × FR: *F* (1, 41) = 0.06, *p* = 0.8117; Fig. 4e). Proactive close following of the robo-beetle was virtually absent in HAB mice (strain: *F* (1, 41) = 45.50, *p* < 0.0001) and unaffected by food restriction (FR: *F* (1, 41) = 0.52, *p* = 0.4765; strain × FR: *F* (1, 41) = 0.02, *p* = 0.8778; Fig. 4f).

### Motivational aspects assessed by operant conditioning

To compare the motivational impact of food restriction between HAB and NAB mice, we tested new cohorts of animals in an operant conditioning task, in which the animals have to work with increasing effort for food (wanting). At the population level, NAB were slightly retarded in acquisition of the operant conditioning task with 2/3 of the NAB, but >90% of HAB reaching the PR protocol within 25 sessions. This nonsignificant line difference implies a slightly higher motivation of HAB to work for food at population level, even though we cannot exclude general learning differences. At individual level, however, if only those mice were considered, which had promoted to PR training, there were no strain differences observable in breaking points (HAB: 40.1 ± 2.0, NAB: 39.9 ± 2.0; *t* = 0.06, d.f. = 17, *p* = 0.9510) and number of target touches (HAB: 251.7 ± 22.6, NAB: 254.4 ± 21.9; *t* = 0.08, d.f. = 17, *p* = 0.9365). Consequently, it is rather unlikely that the line differences in exploration, fear-, and anxiety-related behavior observed before did simply result from different motivational impact of food restriction.

### Principal component analysis

**Fig. 5:**
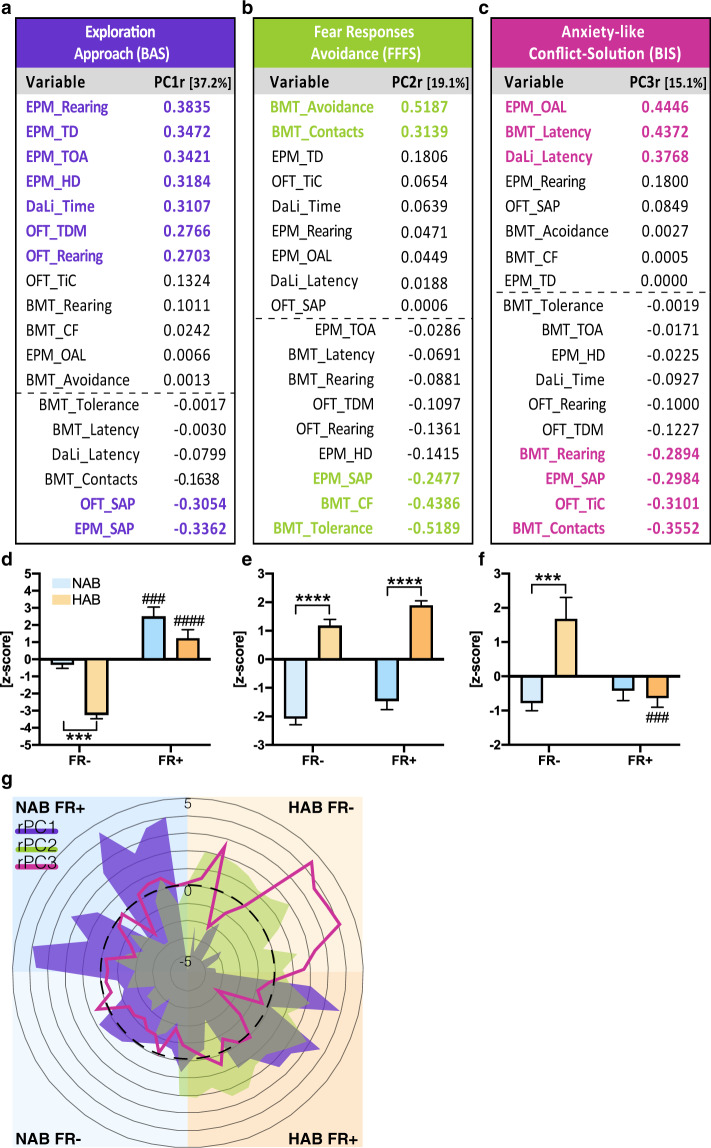
Principal component analysis (PCA). **a**–**c** PCA over the 18 variables from OFT, EPM, DaLi, and BMT (cf. Figs. 1–4) revealed three main rotated principal components (PC1r to PC3r). Behavioral readouts with a loading >0.20 or <−0.20 were highlighted in bold. All variables were weighted according to their loadings and used to calculate individual *z*-scores for each group and PC. Based on the loadings, the components stand for exploration (PC1r), fear-related behavior (PC2r), and anxiety-related behavior (PC3r), respectively, approach behavior (controlled by the Behavioral Activation System, BAS), avoidance behavior (controlled by the Fight/Flight/Freeze system, FFFS), and conflict solving (controlled by the Behavioral Inhibition System, BIS), if translated to the Reinforcement Sensitivity Theory^8,10^. **d**–**f** Impact of food restriction on the *z*-scores for the difference PCs. ###*p* < 0.001, ####*p* < 0.0001 respective FR− group. For further details, see Fig. 1. **g** Spider web plot depicting the individual *z*-scores for the experimental groups and PCs. The black line corresponds to the grand mean. Data points outside the black circle stand for increased approach, avoidance, and anxiety-related behavior; data points within the black circle for decreased approach, avoidance, and anxiety-related behavior. BMT beetle mania task, CF close following, DaLi dark–light transition task, EPM elevated plus maze, HD head dipping, OAL open arm latency, OFT open field test, PC principal component, SAP stretch-attend posture, TD total distance, TDM total distance moved, TiC time in center, TOA time in open arms.

In order to reduce the dimensionality of our data, we performed an unbiased PCA. We obtained three main components, which explained a total of 71. 3% of the variance. Based on the factorial loadings, we classified PC1r as a exploration component, PC2r as fear component, and PC3r as anxiety-related component (Fig. 5a–c). Next, we calculated individual scores for each mouse and component, whereby the variables were weighted by their loadings. Subsequent analyses of those scores by two-way ANOVAs revealed significant strain differences (strain: *F* (1, 41) = 23.79, *p* < 0.0001) for PC1r. FR+ caused a general increase in exploration (FR: *F* (1, 41) = 70.72, *p* < 0.0001) irrespective of the strain (strain × FR: *F* (1, 41) = 3.703, *p* = 0.0613; Fig. 5d).

In case of PC2r, we observed significant strain differences with HAB showing—in general—increased fear-related behavior compared to NAB (*s*train: *F* (1, 41) = 186.2, *p* < 0.0001). FR caused an increase in fear-related behavior (FR: *F* (1, 41) = 7.668, *p* = 0.0084), irrespective of the strain (strain × FR: *F* (1, 41) = 0.04, *p* = 0.8416; Fig. 5e).

In case of PC3r, the a priori strain differences in anxiety-like behavior (strain: *F* (1, 41) = 8.02, *p* = 0.0071) were selectively ameliorated in HAB mice (FR: *F* (1, 41) = 6.08, *p* = 0.0179; strain × FR: *F* (1, 41) = 11.44, *p* = 0.0016; Fig. 5f).

The penetrance of the FR effects within the population of mice became evident, when we considered the individual data (Spider web plot; Fig. 5g).

The outcome of a PCA critically depends on the analytical settings. We decided to rotate the components in order to improve their interpretability. We preferred the varimax rotation to preserve the orthogonality resulting from the PCA. However, a promax rotation resulted in almost the same components, and the individual scores from the varimax rotation are highly correlated with the scores from the promax rotation (PC1r: *r* = 0.9928; PC2r: *r* = 0.9999; PC3r: *r* = 0.9956).

The relatively low ratio (2.5) between subject number (*n* = 45) and readout number (18) seems to limit the generalizability of our findings. However, the needed ratio also depends on the number of components of interest. In this case, we were interested in the first three components only and, therefore, we considered the ratio as sufficient. In addition, to estimate the stability of the results, we performed 5000 PCAs with varimax rotation of the first three components, where we randomly excluded five animals (10% of data) in each run, and calculated the scores for each animal and component. These scores of the subset were correlated with the scores of the whole data, yielding to 5000 correlation coefficients for each component. The correlations for the third component were slightly weaker than for the first two. However, all were extremely strong (PC1r: *M* = 0.999, SD = 0.002; PC2r: *M* = 0.998, SD = 0.003; PC3r: *M* = 0.989, SD = 0.018), thus indicating robust results.

## Discussion

Mice bred for high levels of open arm avoidance (HAB) exhibited elevated anxiety-like (cf. ref. ^14^) and fear-related behavior (cf. refs. ^16,21^), compared to their corresponding controls (NAB). Food restriction (FR+) caused a general increase in exploration and selectively ameliorated anxiety-like behavior in HAB mice, whereas fear-related behavior was even more pronounced. By means of PCA we identified three components, which correspond to the approach behavior, avoidance behavior, and decision-related processes proposed by the Reinforcement Sensitivity Theory^7,8^.

### Food restriction increased foraging behavior in both NAB and HAB mice

In the OFT, HAB mice showed reduced horizontal and vertical exploration of the novel area under basal conditions (FR−). Remarkably, the reduction in locomotor activity was accompanied by an increase in risk assessment behavior (SAP duration). This illustrates the interest of the animals in environmental exploration and precludes fatigue, sleepiness, and lack of motivation as alternative explanation for the reduced locomotion. Instead, changes in behavior shown by food-restricted HAB mice suggests anxiety as major source of hesitant exploration. Food restriction normalized horizontal and vertical exploration. Moreover, it increased the exploration of the unprotected center and reduced risk assessment, which is particularly sensitive to anxiolytic compounds^22–24^. This interpretation is further supported by data from the EPM and DaLi. In the EPM, food restriction caused an increase in open arm exploration and head dipping, which coincided with a general increase in exploration. This time, however, the effects could be observed in both HAB and NAB. This might be explained by a higher anxiety load of the EPM as compared to the OFT that may also affect control mice. In the DaLi, food restriction increased the exploration of the light compartment again selectively in HAB mice.

### Food restriction enhances active fear responses

The BMT confronts mice with an erratically moving robo-beetle to measure active fear responses. HAB mice showed exaggerate avoidance behavior, which confirmed previous observations^16^. Food restriction further enhanced avoidance behavior in HAB (and NAB) mice, indicative of fear-promoting effects. This is in striking contrast to the amelioration of anxiety-related behavior and the increase in exploratory drive, which could also be observed in the BMT during basal exploration of the setup (i.e., before introduction of the robo-beetle).

### Food restriction and the Reinforcement Sensitivity Theory

Changes in anxiety-related behavior invariably coincided with changes in exploratory drive^25,26^. To disentangle both domains and to “correct” for multiple testing, we reduced the dimensions of the manifold behavioral readouts using PCA (cf. ref. ^27^). We obtained three main principal components, which explained together >71.3% of the total variance.

Most exploration-related readouts loaded on PC1r, including data on horizontal and vertical locomotor activity, but also open arm time in the EPM, time in the light compartment in the DaLi and risk assessment (SAPs) shown in the open field and the EPM. The latter readouts are commonly seen as measures of anxiety-related behavior^28–32^. This illustrates the proximity of exploration and “standard” anxiety-related readouts and the difficulty to disentangle the two domains^25,31^.

PC2r covered most of the fear-related measures obtained in the BMT. Both, number of contacts with the robo-beetle and avoidance positively loaded on the factor. The relationship between the two variables does not simply reflect the higher incidence of contacts in HAB, since we normalized avoidance to the number of contacts. Instead, it is conceivable that increased freezing upon longer distance between mouse and robo-beetle and the occurrence of flight behavior upon close proximity of the robo-beetle account for this coincidence. Following this logic, we can assume that food restriction does not change the perception of defensive distance (if not even sharpens it).

PC3r was dominated by anxiety-related readouts from the OFT (i.e., time in center), EPM (latency to first open arm entrance), DaLi (latency to enter the light compartment), and BMT (latency to reach the other side of the arena during baseline). Remarkably, PC3r was devoid of any significant loadings by measures of locomotor activity. Also, loadings of time on open arms (EPM) or in the light compartment (DaLi)—the “standard readouts” for anxiety-related behavior—were missing. Apparently, PC3r primarily contains readouts, which are indicative of decision processes, such as latencies until the first entry into potentially threatening environments. This is in line with theories, which consider conflicts between competing goals and decision-making processes as essential components of anxious states^1,2,28^.

There have been numerous studies employing statistical tools, such as factor analysis/PCA for the isolation of different dimensions in exploratory behavior. They either used this statistical approach for a single task^33–36^ or collapsed the analyses over multiple test situations^27,37–40^. Most studies remained at the descriptive level and did not attempt to integrate the findings into theoretical frameworks. The present study, in contrast, changed the exploratory drive of the animals by comparing food-restricted with ad libitum-fed mice. Moreover, we studied exploratory behavior in different test situations with more (e.g., EPM) or less (e.g., BMT) ambiguous threat confrontation. This allowed us to consider both state emotional phenotypes (as observed in a given test situation) and trait phenotypes (which should result in test-overarching phenotypes).

The three components identified by PCA can be interpreted best by the three independent systems of the Reinforcement Sensitivity Theory, with PC1r dominated by the approach-controlling BAS, PC2r the avoidance-controlling FFFS, and PC3r the conflict-solving BIS^8,10^. Comparison of the *z*-scores for the different experimental groups and components revealed that food restriction caused a general activation of approach behavior (i.e., the BAS), potentiated avoidance behavior (i.e., the FFFS), and selectively reduced anxiety-related decision making (controlled by the BIS) in HAB mice.

According to the Reinforcement Sensitivity Theory, the BAS (or “Let’s go for it!” system^9,12^) is defining reward sensitivity^7–10^. Accordingly, food restriction causes an increase in foraging behavior as reflected by increased horizontal and vertical exploration on expenses of decreased risk assessment (PC1r). This increase could be observed in both NAB and HAB mice. Without additional motivation (i.e., with food ad libitum), HAB mice showed very low levels of exploration. With the help of an operant conditioning task in which animals have to work for food (PR4) we could exclude that HAB mice, in general, show reduced reward sensitivity compared to NAB mice. Therefore, without food restriction, precautious behavior seems to outbalance exploratory drive in these animals.

The FFFS (or “Get me out here!” system^9^) is thought to define punishment sensitivity and, thus, mediates defensive reactions to aversive stimuli. The higher scores shown by HAB mice resonate well with numerous other studies, which described increased conditioned (e.g., passive avoidance^20^, auditory-cued, and contextual fear^20,41^, mediated by the BIS) and unconditioned fear (e.g., avoidance of predator scent^42^). Unexpectedly^15^, food restriction not only failed to revert the phenotype of HAB mice, but even further enhanced it. Likely, the increased motivation to forage for food has different consequences on fear responses than situations, in which mice could satisfy their desire for food. Richard Palmiter and colleagues could identify a class of neurons in the parabrachial nucleus, which is activated by threatening stimuli and silenced by food intake. Artificial silencing of those neurons reduced the expression of conditioned fear^43^, thus suggesting them as a master switch between defensive responding vs. food intake.

The BIS (or “Watch out, be very careful!” system^9^), finally, is responsible for the resolution of goal conflicts. With the increase in motivation, food-restricted HAB mice more readily explore aversive environments (e.g., latencies to enter the open arms of the EPM or center of the open field). The effects of food restriction have been limited to HAB mice and, thus, to animals with exaggerated trait anxiety. Starving mice on the C57BL/6 background also showed decreased anxiety in conflict-based tasks (e.g., EPM and Pavlovian food challenge test) with a prominent role of hypothalamic agouti-related peptide-expressing neurons^44^. Future studies have to explore the involvement of this class of neurons and the role of the peripheral anxiolytic “hunger hormone” ghrelin^45^ in food-restricted HAB mice.

It is of note that several points limit the interpretation of our findings: first, our conclusions are based on a limited number of animals. Second, we focus on the three main components of the PCA. Third, we only considered the Reinforcement Sensitivity Theory when interpreting those three components, while disregarding other personality theories such as Eysenck’s arousal/activation theory of Introversion-Extraversion and Neuroticism^46–48^. Fourth, future studies have to assess the impact of classical anxiolytics on the different behavioral components.

## Conclusions

In conclusion, we demonstrate that approach, avoidance and conflict-solving behavior are experimentally and statistically dissociable dimensions in foraging mice. Future preclinical studies on anxiety-related behavior should replace simplistic “standard” behavioral readouts (e.g., open arm time for the EPM test), and their anthropocentric interpretation by more sophisticated approaches and testing of the animals with different motivational states. Our data are well explained by the Reinforcement Sensitivity Theory, which deserves broader consideration in future preclinical and clinical studies on the neuronal basis of emotions, motivations, personality traits, and psychopathology.
